# Prismatic Adaptation Induces Plastic Changes onto Spatial and Temporal Domains in Near and Far Space

**DOI:** 10.1155/2016/3495075

**Published:** 2016-02-14

**Authors:** Ivan Patané, Alessandro Farnè, Francesca Frassinetti

**Affiliations:** ^1^Department of Psychology, University of Bologna, 40127 Bologna, Italy; ^2^ImpAct Team, Lyon Neuroscience Research Centre, INSERM U1028, CNRS UMR5292, 69676 Lyon, France; ^3^UCBL, Lyon I University, 69100 Villeurbanne, France; ^4^Hospices Civiles de Lyon, Neuro-immersion & Mouvement et Handicap, 69676 Lyon, France; ^5^Fondazione Salvatore Maugeri, Clinica del Lavoro e della Riabilitazione, IRCCS, Istituto Scientifico di Castel Goffredo, 46042 Mantova, Italy

## Abstract

A large literature has documented interactions between space and time suggesting that the two experiential domains may share a common format in a generalized magnitude system (ATOM theory). To further explore this hypothesis, here we measured the extent to which time and space are sensitive to the same sensorimotor plasticity processes, as induced by classical prismatic adaptation procedures (PA). We also exanimated whether spatial-attention shifts on time and space processing, produced through PA, extend to stimuli presented beyond the immediate near space. Results indicated that PA affected both temporal and spatial representations not only in the near space (i.e., the region within which the adaptation occurred), but also in the far space. In addition, both rightward and leftward PA directions caused opposite and symmetrical modulations on time processing, whereas only leftward PA biased space processing rightward. We discuss these findings within the ATOM framework and models that account for PA effects on space and time processing. We propose that the differential and asymmetrical effects following PA may suggest that temporal and spatial representations are not perfectly aligned.

## 1. Introduction 

The constructs of time and space saturate our discourses, as the two experiential domains are, of course, fundamental to how we interact with and organize our environment. Considerable empirical evidence has revealed interactions between time and space in both brain and behavior, from low-level perception to high-level linguistic processes. In this respect, Walsh's prominent theory (well known as A Theory of Magnitude, ATOM) [1] has tried to account for these systematic interdomain influences. As posited by this theory, time and space, along with other forms of magnitude, may share a single cross-domain metric system in the brain, in particular within regions of the parietal cortex [1, 2]. A distributed and integrated network of other areas, such as the prefrontal cortex, are presumably involved in the magnitude system, even if the cross-domain magnitude representations seem to be specially coded by the parietal regions [2]. Thus, temporal events and spatial extents may share a representational and neural format, such that two events are separated by a particular duration in the same way as two locations are separated by a particular distance.

In addition to this neural model, at the behavioral level one of the most well-established interactions between space and time is captured by the Spatial Temporal Association of Response Codes effect (STEARC effect): temporal concepts, such as “before” versus “after,” are spatially associated with horizontal locations [3–7]. Converging evidence of time-space interactions has therefore supported the proposal that time is represented spatially along a horizontal mental time line (“MTL”) [8, 9]. Hence, it has been suggested that humans experience time as flowing along a mental horizontal line, so that smaller magnitudes (intervals) of time are associated with the left part of the line and larger magnitudes (intervals) are associated with the right part of the line. Accordingly, the duration of stimuli presented in the left space is underestimated relative to that of stimuli presented in the right space, which tends to be overestimated [10, 11].

In line with the finding of a spatial representation of time intervals in ascending order from left to right, a “distortion” of time perception is observed following leftward or rightward attentional shifts created through prismatic adaptation (PA) procedure. Broadly speaking, PA is a well-known sensorimotor technique for investigating cerebral plasticity in neurologically healthy individuals by inducing a relatively transient shift of spatial attention [12–16]. In addition, leftward- and rightward-deviating prisms affect performance on a variety of temporal tasks. Previous research has indeed reported that PA is able to modulate systematically the spatial representation of time in healthy individuals [11, 17, 18]. In these studies, participants performed a time (duration) reproduction task and/or a time (duration) bisection task, before and after leftward (LPA) and rightward (RPA) prismatic adaptation. According to the above-recalled left-to-right organization of the spatial representation of time, PA caused an opposite and symmetrical modulation on time processing: LPA produced an overestimation of time durations, whereas RPA produced an underestimation of time durations. These findings have thus demonstrated that manipulating spatial representations can shape biases in the estimation of time durations.

Somewhat similarly, biasing attention orientation by means of PA alters performance, not only in temporal, but also in visuospatial tasks. So, for instance, in a distinctive visuospatial task, the line bisection, young individuals typically show pseudoneglect: they judge the middle of the horizontal line to the left of the true center [19–23]. More interestingly, following adaptation to leftward prism (LPA) participants shift their midline judgments to the right, thus reducing or cancelling their leftward bias at baseline [16, 24–27]. It is worth noting that most studies have demonstrated a significant modulation after LPA, but no effect following RPA on visuospatial processing [16, 24–29].

In the bine bisection task, however, such a pseudoneglect phenomenon appears to be limited to stimuli presented close to the body, with bias shifting to the right of the true center as viewing distance increases [23, 30–34]. Specifically, individuals show a leftward bias (i.e., pseudoneglect) within near space and a rightward bias in far space, and the point of transition from the left-to-right bias can be manipulated by extending or contracting the participant's reach [32, 34]. This pattern, systematically related to reach capabilities, has been interpreted as evidence of distinct representations of space, which are encoded on the basis of action potentiality [35–37]. This interpretation is in line with the proposal that pseudoneglect is usually found in the space near the body where individuals can act upon directly by the use of the arm, whereas rightward bias is observed where the line to be bisected is presented in the space far from the body, beyond the space with immediate action potential [32–34]. Indeed, the results of neurophysiological [23, 38–41], neuropsychological [42–44], and neuroimaging studies [45–48] converge suggesting that, on the basis of the potential actions that can be performed, the space close to the body (i.e., near space) and the space that lies just beyond reach (i.e., far space) are represented differently by the brain (for reviews, see [48–51]).

Based on the abovementioned evidence indicating tight relationships between attentional mechanisms and representations of space and time, our study was designed to explore, for the first time within the same experimental design, the effect of PA in both near and far space on both spatial and temporal tasks. So far indeed, previous research has investigated the effect of the shift of spatial attention on time processing in the near, but not in the far space.

For the purpose of the study, the landmark task, which is a nonmanual, perceptual variant of line bisection, was used to measure the visuospatial bias caused by PA. We chose this paradigm in order to minimize the influence of motor factors on bisection judgments and to slow down the deadaptation process after PA procedure [16, 22, 24, 52–54]. In this task, participants are required to make forced-choice responses according to whether they perceive a vertical transector to be closer to the left or right end of a horizontally displayed line.

The time bisection task was instead used to measure the modulation on the spatial representation of time caused by PA. The paradigm is a well-established task in which participants classify whether a series of stimuli are closer in duration to a “short” reference or to a “long” reference duration [55–57]. In order to compare the temporal and spatial changes induced by PA, we needed similar stimuli to be employed in the two tasks. To this end, in the landmark paradigm stimuli were lines pretransected at one of different locations and were presented for 2000 ms. Similarly, in the time bisection paradigm stimuli were lines pretransected at veridical center and had one of different durations. Finally, to answer the question of whether PA aftereffects measured by the landmark and time bisection tasks would extend to stimuli presented in the far space, all subjects were submitted to the two tasks at two different distances: 60 cm (within arm's reach, i.e., in near space) and 120 cm (outside arm's reach, i.e., in far space).

## 2. Materials and Methods

### 2.1. Participants

Sixty-four healthy volunteers (46 females, mean age = 23.78; SD = 2.13 years) participated in the study (mean education = 16.27; SD = 1.61 years). The participants had no self-reported history of neurological or psychiatric diseases, had normal or corrected-to-normal vision, and were right-handed according to self-report and as assessed by administration of the Edinburgh Handedness Inventory (mean = 78.29; SD = 17.51) [58]. They were naïve as to the experimental hypotheses being tested and gave informed consent to take part in this study, which was approved by the local ethics committee and conducted in accordance with the ethical standards of the 2008 Declaration of Helsinki.

### 2.2. Design

All data were collected in a noise-attenuated room. The experiment consisted of two blocks: one before and one after a PA session. The preadaptation block included the landmark and the time bisection tasks; then participants performed a single session of PA. In the postadaptation block they repeated the landmark and the time bisection tasks five minutes after prisms removal. Within each block, the landmark and time bisection tasks were performed at two viewing distances: near space (60 cm, i.e., monitor screen placed within arm's reach) and far space (120 cm, i.e., monitor screen placed beyond reaching). The order of the tasks and the two viewing distances was counterbalanced across participants. Participants were asked to keep their eyes closed between the tasks and to rest their hands on their thighs during the whole experiment, except during the PA session. The experimenter ensured that the subject's body position remained constant throughout the experiment. All phases of the experimental session are described in details in the following paragraphs.

### 2.3. Landmark Task

The paradigm represented a computerized version of the landmark task. Participants were comfortably seated directly in front of the monitor screen, with lights extinguished to reduce external visual cues. Their midsagittal planes were aligned with the monitor screen. Viewing distance was also held constant using a chin-rest.

#### 2.3.1. Stimuli

Stimuli were presented using the E-Prime software package (Psychology Software Tools, USA) on a LDC monitor (17 inches, 1280 × 1024 pixel resolution and 75 Hz refresh rate) controlled by a MacBook Pro laptop. Stimuli were white pretransected lines of 100% contrast displayed on a black screen positioned 60 cm (near space) or 120 cm (far space) from the participant's eyes. The size of horizontal line stimuli was adjusted across viewing distances to ensure the retinal angle subtended remained constant at 19.03° of VA (visual angle) in width by 3.23° of VA in height. Horizontal lines stimuli were centered with respect to both the midsagittal plane of each subject and the computer screen. Lines were pretransected at the true center (i.e., 0.00°) and at ±0.1°, ±0.2°, ±0.3°, ±0.4°, and ±0.5° of VA toward the left and right of the true center. Transectors were 1.53° in vertical length and 0.08° thick. Each of the 11 different pretransected lines was presented eight times in a random order, yielding a total of 176 trials, 88 in the near space, and 88 in the far space. The order of presentation in near and far space was counterbalanced across participants.

**Figure 1:**
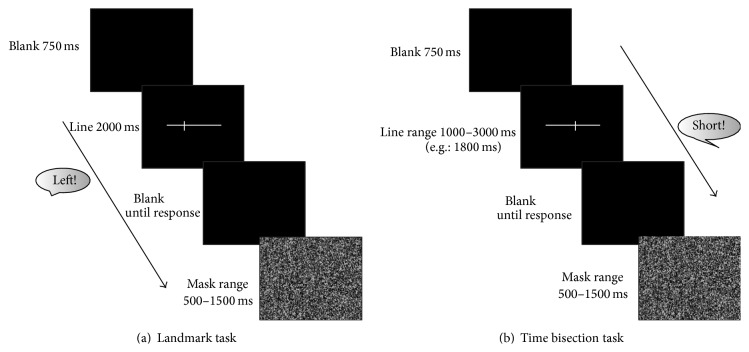
Graphical representation of sequence of events in each trial for the two tasks. (a) Landmark task. Following 750 ms presentation of a blank, pretransected lines were presented for 2000 ms before reappearance of the blank on the screen until the subject responded. Lines were pretransected at 1 of 11 locations ranging symmetrically from −0.5° to +0.5° of visual angle (distance between transector locations = 0.1°) and including veridical center. After the participant's response (the transector is closer to the “right” or to the “left”), a black-and-white patterned mask was presented at random duration (range 500–1500 ms) before the next trial was presented. (b) Time bisection task. Following 750 ms presentation of a blank, pretransected lines were presented before reappearance of the blank until the subject responded. Lines were pretransected at veridical center and had 1 of 11 different duration ranging from 1000 to 3000 ms at 200 ms intervals. After the participant's response (the duration of the line is “short” or “long”), a black-and-white patterned mask was presented for a random duration (range 500–1500 ms) before the next trial.

Trials began with the presentation of a blank (black background) for 750 ms, after which the line stimulus was presented. Each pretransected line was displayed for 2000 ms and then replaced by a blank (black background), which remained visible until a response was made. After the response, a black-and-white patterned mask stayed on the screen for a random duration (range 500–1500 ms) before the subsequent trial was presented (see Figure 1).

#### 2.3.2. Procedure

The task was a two-alternative forced-choice paradigm and consisted of the verbal classification of pretransected lines that appeared centrally displayed on the monitor screen. On each trial, participants were asked to fully inspect each pretransected line and to judge which end of the line the transector was closer to. Thus, participants were instructed to verbally classify each prebisected line as “left” if the transector was perceived as being closer to the left end of the line and as “right” if they perceived it to be closer to the right end. To ensure that participants understood the task instructions, a practice session was administered before the experimental task. In this practice session participants had to classify five lines pretransected at −0.5° as “left” and five lines pretransected at +0.5° as “right,” all presented in a random order. Participants received feedback only in these ten practice trials, which was repeated until they had reached at least 80% of accuracy. All participants reached such level of accuracy with the first practice session. Then, they were submitted to the experimental task in which all the possible eleven prebisected lines were displayed in random order. Participants were required to make a left/right-forced-choice and were told to make their best guess if they were unsure. They were also instructed to respond as accurately and as quickly as possible, without receiving any feedback. The experimenter seated behind participants (at least 1 m) and recorded participants' verbal responses by pressing one of two keys on a keyboard (“Q” for left and “P” for right). The landmark task took approximately twelve minutes to complete.

#### 2.3.3. Data Analysis

In order to obtain an objective measure of perceived line midpoint in each condition for each participant, cumulative Gaussian functions were fitted to the proportion of “right” responses (i.e., when subjects judged the transector as being at the right of the true center) given by each participant as a function of the position of the 11 transector positions. The estimate of the point at which the psychometric function cuts the 50% of “right” responses indicates the point of subjective equality (PSE). An increase of “right” responses after PA, as compared to baseline, induces a decreased PSE, reflecting a relative shift toward the left of the perceived midline. On the contrary, an increase of “left” responses after PA, as compared with performance at baseline, induces an increased PSE, reflecting a relative shift to toward the right. So, the PSE allows us to detect any rightward (leftward) spatial shifts induced by PA on midpoint judgments in near and far spaces.

### 2.4. Time Bisection Task

The experimental procedure of the time bisection task was similar to the landmark paradigm, with the following exceptions.

#### 2.4.1. Stimuli

Stimuli were lines, presented on the monitor, always pretransected at the true center (i.e., 0.00° of VA). Lines had different durations that were linearly spaced from 1000 to 3000 ms at 200 ms intervals (i.e., 1000, 1200, 1400, 1600, 1800, 2000, 2200, 2400, 2600, 2800, and 3000 ms). The size of the pretransacted lines and the monitor distance were the same of the landmark task in order to keep constant the visual angle at the two viewing distances (60 cm and 120 cm). Eight trials for each of the 11 different intervals were randomly presented, yielding a total of 176 trials, 88 in the near space and 88 in the far space. Trials began with the presentation of a blank (black background) for 750 ms, after which the line stimuli were presented. One of 11 lines with different durations (range 1000–3000 ms) was displayed and then replaced by a blank (black background), which remained visible until a response was made. After the response, a black-and-white patterned mask stayed on the screen for a random duration (range 500–1500 ms) before the subsequent trial was presented (see Figure 1).

#### 2.4.2. Procedure

The time bisection task consisted of the verbal classification of a series of pretransected lines that were displayed for different durations at the center of the computer screen. Participants were instructed to verbally judge (forced-choice) whether the duration of each line was “short” or “long” with respect to previously acquired pair of reference durations (1000 ms and 3000 ms). Before administering the experimental task, the practice session served to familiarize participants with the two reference durations presented in random order. In this session ten practice trials were displayed and participants had to classify five intervals of 1000 ms as “short” and five intervals of 3000 ms as “long.” Feedback was given on accuracy only for these ten practice trials. All participants reached at least 80% of accuracy with no more than two practice sessions. After the experimenter had ensured participants were confident with the practice session, all the eleven lines with different durations were presented in random order during the experimental task. Participants were required to classify each line duration as “short” or “long” and to make their best guess if they were unsure. The experimenter was seated behind the participants (at least 1 m) and recorded participants' verbal responses by pressing one of two keys on a keyboard (“Q” for “short” and “P” for “long”). The task in the near and in the far space took approximately fourteen minutes to complete.

#### 2.4.3. Data Analysis

A psychophysical response function was created for each participant by calculating the proportion of “long” responses for each of the 11 line durations. The data were fit with cumulative Gaussian function, whose mean (50% point) indicated the “Point of Subjective Equality” (PSE). The PSE is the duration at which a participant is equally likely to classify the stimuli as short or long. For each participant, the PSEs were separately calculated in each condition.

An increase of “long” responses after PA, as compared to baseline performance, induces a decreased PSE, reflecting a relative shift towards overestimation of temporal midpoint (i.e., durations are perceived being longer with respect to before PA). Conversely, an increase of “short” responses after PA, as compared with performance at baseline, induces an increased PSE, reflecting a relative shift towards underestimation of temporal midpoint (i.e., durations are perceived shorter).

Accordingly, the PSE allows us to observe whether PA caused a bias in their temporal judgment towards either an underestimation or an overestimation of durations in near and far spaces.

### 2.5. Prism Adaptation

The procedure, stimuli, and material conformed to well-established PA described in Frassinetti et al.' study [11]. Subjects were submitted to either the rightward prismatic goggles inducing a leftward aftereffect (RPA group, *n* = 32, 23 females; mean age = 23.66; SD = 1.91) or the leftward prismatic goggles inducing a rightward aftereffect (LPA group, *n* = 32; 23 females; mean age = 23.91; SD = 2.35). During PA subjects were seated at a table in front of a box (height = 30 cm, depth = 34 cm at the center and 18 cm at the periphery, width = 72 cm) that was open on the side facing the participants and on the opposite side, facing the experimenter. The box consisted in a Plexiglas panel erected in a vertical and semicircular position and graduated by vertical lines spaced by 1° of VA. The experimenter placed a visual target (a pen) at the distal edge of the top surface of the box, in one of three possible positions (randomly determined on each trial): a central position (0°), 21° to the left of the center, and 21° to the right of the center. Subjects were asked to keep their right hand at the level of the sternum and to point toward the pen using the index finger of the same hand as quickly and as accurately as possible. Participants were also instructed to make a ballistic movement and to correct any errors on the successive movement. Participants could not see their hand when it was at the starting position and during the first third of the pointing movement to ensure effective adaptation. The experimenter recorded the end position of the subject's pointing direction. The pointing task was performed in three experimental conditions: preadaptation, adaptation, and postadaptation. In the preadaptation condition, the subjects performed two types of trials (total of 60 trials). On half of the trials, their pointing was visible to them (as in the adaptation condition). On the other half, the subjects were required to look at the target and then to point at it with their eyes closed, so that the pointing was not visible at any stage of the movement (i.e., invisible pointing as in the postadaptation condition, open-loop pointing). In the adaptation condition (total of 90 trials), the subjects performed the task while wearing prismatic lenses that induced a 10° shift of the visual field to the left (LPA) or to the right (RPA). Participants could see the trajectory of their arm (i.e., visible pointing). In the postadaptation condition, immediately after prisms removal, the subjects were required to look at the target and make their pointing movements with their eyes closed as in the preadaptation condition (i.e., open-loop pointing; 30 trials). The adaptation procedure took approximately 15–20 min to complete.

## 3. Results

### 3.1. Landmark and Time Bisection Tasks

We focused our results exclusively on the mean PSEs yielded by these tasks. Therefore, for each participant the PSEs in the temporal and spatial tasks were separately calculated for each condition.

Kolmogorov-Smirnov tests separately conducted for each group and experimental condition revealed that data were normally distributed (i.e., the tests of normality gave nonsignificant results).

In order to explore PA effects on temporal and spatial judgments in the near and the far space, we ran a multivariate analysis of variance for repeated measures. MANOVA would supposedly reduce the experiment-wise level of Type I error compared to several separate analyses and allows for taking into account potential correlations among the dependent variables of different nature (here expressed in milliseconds and degrees of visual angle).

The MANOVA was performed on the average PSEs measured in the landmark and the time bisection tasks, considering as within-subject factors* space* (near, far) and* session* (pre-PA, post-PA), and as between-subject factors* prisms* (LPA, RPA).* Post hoc* paired *t*-tests with the appropriate Bonferroni correction were conducted, where necessary, unless otherwise specified. Partial eta squared and Cohen's *d* are used to report effect size. As preliminary analyses failed to find any significant effect of age, education, or Edinburgh scoring, these factors are not considered further here.

The multivariate analysis revealed a significant multivariate main effect for* space*, Wilks' *λ* = .823, *F*(2, 61) = 6.55, *p* = .003, and *η* ^2^_*p*_ = .18. Overall in the landmark task,* post hoc* analysis showed a negative PSE value in the near space, indicating that participants perceived the middle of the line towards the left of the true center (−0.026°), and a positive PSE value in the far space, indicating that participants perceived the middle of the line towards the right (0.016°, *p* < .001, *d* = 0.42). However, in the time bisection task, it must be pointed out that the difference between the mean PSE in near (1891 ms) and far space (1926 ms) was not significant (*p* > .05).

**Figure 2:**
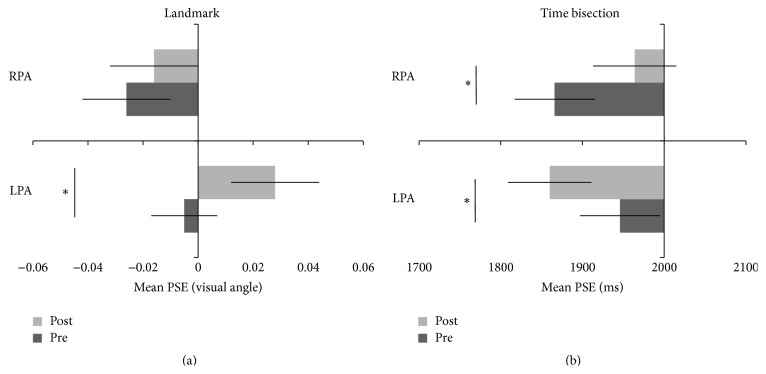
Point of subjective equality judgments (PSE) as a function of prism group (LPA and RPA) and session before (dark gray) and after PA (light gray). Landmark task (a): the line transector is judged (in visual angle) as nearer to the left (negative values) or to the right (positive values). After LPA the line transector is judged as nearer to the right compared to before PA (positive values). Time bisection task (b): interval duration (in millisecond) is classified as short (<2000 ms) or as long (>2000 ms). After LPA the time interval is classified as longer compared to before, whereas after RPA the time interval is classified as shorter compared to before PA. Bars represent the average point of subjective equality for spatial and temporal judgments, respectively, ± standard error (SEM). ^*∗*^ *p* < .05.

More important for the aim of the study, the MANOVA found a significant multivariate interaction effect* session* by* prisms*, Wilks' *λ* = .667, *F*(2, 61) = 15.21, *p* < .001, *η* ^2^_*p*_ = .33 (see Figure 2). In the landmark task, there was a significant rightward shift of the mean PSE after leftward-displacing prismatic adaptation with respect to before (post-LPA 0.028°; pre-LPA −0.005°, *p* = .008, Bonferroni corrected *p* = .047, and *d* = 0.45). On the contrary, prismatic adaption with rightward visual displacement did not affect the spatial judgments, as measured by the PSE (post-RPA = −0.016°; pre-RPA = −0.026°, *p* > .05).

By contrast, both right- and left-deviating prisms biased symmetrically the PSEs in the time bisection task. Indeed, LPA produced an overestimation of time durations as compared to a pre-adaptation baseline (post-LPA = 1860 ms; pre-LPA = 1946 ms, *p* = .001, Bonferroni corrected *p* = .006, and *d* = 0.6), whereas RPA produced an underestimation of time durations (post-RPA = 1964 ms; pre-RPA = 1866 ms, *p* < .001, Bonferroni corrected *p* = .001, and *d* = 0.74). Importantly, the symmetrical effect of LPA and RPA did not seem to be driven by a difference between the two prism groups at the baseline in the time bisection task, since the PSE measures before prismatic adaptation did not differ (Bonferroni corrected *p* = .773, *d* = .28, and power = .88).

Since the multivariate three-way interaction was not significant, Wilks' *λ* = .952, *F*(2, 61) = 1.55, *p* = .221, and *η* ^2^_*p*_ = .05, in the landmark task the spatial modulation following adaption procedure was present in both near (pre-LPA = −0.028°; post-LPA = 0.017°) and far space conditions (pre-LPA = 0.017°; post-LPA = 0.038°) for the leftward-deviating prism group. In contrast, no significant change emerged either in near (pre-RPA = −0.042°; post-RPA = −0.051°), or in far spaces (pre-RPA = −0.009°; post-RPA = 0.018°) for the rightward-deviating prism group. Remarkably, given that the symmetrical effects of PA on the time bisection task did not vary depending on the location of stimuli, temporal modulation caused by prisms was evident for the LPA group, in both near (pre-LPA = 1947 ms; post-LPA = 1844 ms) and far space conditions (pre-LPA = 1944 ms; post-LPA = 1876 ms), and for RPA group, in near (pre-RPA = 1837 ms; post-LPA = 1937 ms) and well as in far space conditions (pre-RPA = 1895 ms; post-RPA = 1991 ms).

Besides this, as suggested by the anonymous reviewers, we checked whether the MANOVA results described above might have been determined by differences in baseline values. We computed unpaired *t*-tests (both uncorrected and Bonferroni corrected for multiple comparisons) in each experimental task, comparing the mean baseline PSEs between the two prism groups in the near and far conditions. No significant difference emerged, either in landmark or in temporal judgments, prior to the adaptation procedure for the two PA groups (all *p*s > .05).

Finally, a series of Pearson's correlation analyses was performed to assess for possible relationships between the PSEs at baseline in the landmark and time bisection task for either prisms direction. Additionally, a series of correlations was conducted to assess the relationship between the effects of PA on temporal and spatial PSEs by comparing the measures in the two tasks before and after RPA and LPA session. No significant correlation was found (all *p*s > .05).

Taken together these data indicate that, first, spatial judgments were influenced by the space wherein the stimulus to be bisected had been presented (near or far space). More interestingly, the midpoint judgments in the spatial and temporal bisections were affected in a different fashion by PA, regardless of the space where the stimuli had been displayed. Namely, only LPA induced a rightward shift in the perceived midpoint of the line, whereas both RPA and LPA biased spatial representation of time toward an underestimation and overestimation, respectively. The absence of any interaction involving the* space* factor shows that the two different spatial representations (near and far space) did not enforce any differences in the shifts produced by PA, as indexed by the PSEs measured in the temporal and landmark tasks. That is, the effects of prismatic adaptation procedures on time and spatial processing occurred in both near and far spaces in a similar manner.

### 3.2. Prismatic Adaptation

To make sure that any potential differences in time and space estimation before and after prism session were due to PA, we assessed the presence of both error reduction (i.e., the tendency to compensate for prism-induced error in pointing during PA) and aftereffects (i.e., the subsequent tendency to point in the direction opposite to the visual displacement induced by prisms, after goggles removal). Pointing displacement measures (expressed as degrees of visual angle, VA) carry a negative sign (−) when directed to the left and a positive sign (+) when directed to the right with respect to the target actual location.

To demonstrate the presence of pointing errors, in the first trials, and of error reduction, in the last trials of PA condition, participant's pointing performance during preadaptation and adaptation condition was compared. If prism adaptation procedures were effective, a difference should be found between the first trials of the adaptation condition and the preadaptation condition, but no difference should be found between the last trials of the adaptation condition and the preadaptation condition (error reduction).

In order to verify if participants showed an error reduction as they adapted to the prisms, an ANOVA was performed on the mean pointing measures, with* prims* (LPA, RPA) as a between-subject factor and* condition* (preadaptation measures, first three trials of the adaptation measures, and last three trials of the adaptation measures) as a within-subject factor. The ANOVA was followed by* post hoc* (two-tailed) *t*-tests for which the appropriate Bonferroni correction was applied for each prism group. Effect size is indicated as partial eta square or Cohen's *d*.

The ANOVA revealed a significant main effect of the between-subject factor* prisms, F*(1,62) = 572.92, *p* < .001, and *η* ^2^_*p*_ = 0.90 (LPA = −2.163°; RPA = 2.174°). More interestingly, the interaction between* prisms* and* condition* was significant,  *F*(2,124) = 604.83, *p* < .001, and *η* ^2^_*p*_ = 0.91. Pointing error was significantly greater in the first three trials of the adaptation (LPA = −5.872°; RPA = 6.274°) than in the preadaptation (LPA = −0.207°, *p* < .001, Bonferroni corrected *p* < .001, and *d* = 3.41; RPA = 0.049°, *p* < .001, Bonferroni corrected *p* < .001, and *d* = 3.16) but did not differ between the last three trials of the adaptation and the preadaptation values (LPA = −0.410°, RPA = 0.198°, both *p*s > .05). This provided a profile of the direct effects of prismatic displacement, which reflects trial-by-trial error reduction.

To test for the presence of aftereffect, open-loop pointing measures were compared between the postadaptation and the preadaptation conditions. If PA produced a visuomotor bias opposed to deviation induced by prism, a leftward (rightward) error during open-loop pointing should be found when right (left) prismatic goggles have been removed. We conducted an ANOVA with* prisms* (LPA, RPA) as the between-subject factor and* condition* (preadaptation and postadaptation) as the within-subject factor, in order to test this prediction.

The main effect of* prisms, F*(1,62) = 321.81; *p* < .001; *η* ^2^_*p*_ = 0.84, and its interaction with* condition, F*(1,62) = 599.28; *p* < .001; *η* ^2^_*p*_ = 0.90, were significant. The open-loop pointing measures in the postadaptation condition differed from the open-loop pointing measures in the preadaptation in RPA (pre-RPA = −0.025°, post-RPA = −4.509°, *p* < .001, and Bonferroni corrected *p* < .001) as well as in LPA (pre-LPA = −0.396°, post-LPA = 4.517°, *p* < .001, and Bonferroni corrected *p* < .001).

Since the sign of the sensorimotor aftereffects depends upon the direction of the prisms, we also assessed whether the amount of sensorimotor adaptation was similar for the two groups by submitting the absolute value of the shift to a repeated-measures mixed ANOVA with* prisms* (LPA, RPA) as a between-subject factor and condition (pre- and post-PA) as a within-subject factor. In this ANOVA only a main effect of condition emerged, *F*(1,62) = 334.91; *p* < .001; *η* ^2^_*p*_ = 0.84 (pre-PA = 0.732, post-PA = 4.513). This analysis revealed no interaction* prisms* by* condition*, suggesting that both PA groups were equally adapted and that the amount of sensorimotor adaptation was comparable in the two prism groups.

Finally, to assess whether there was any correlation between an individual participant's shift in PSE and the amount of sensorimotor aftereffects or error reduction in pointing, we computed separate Pearson correlation analyses between the absolute value of aftereffect or error reduction and subject's performance in the time bisection and the landmark task for each prism direction. None of these correlations was significant (all *p*s > .05).

In sum, data analysis on PA procedure demonstrated that both groups compensated, during prism adaptation, for prism-induced spatial errors in pointing (error reduction) and that, after prisms removal, they pointed in the direction opposite to the visual displacement induced by prism (aftereffect).

## 4. Discussion 

The main purpose of this investigation was to examine whether the plastic effects on spatial and temporal processing following rightward and leftward prismatic adaptation are generalizable across near and far space. Given a considerable body of evidence suggesting, at least, two independent representations of space based on action potentiality, here we went on to ask whether temporal and spatial modulations induced by PA extend beyond the space near the body. Accordingly, two are the main results of the present study. The first main finding is that manipulation of PA-induced effects influences temporal and spatial judgments not only in the immediate spatial region within which the adaptation occurs (near space), but also in the sector of the space farther away and beyond arm's reach (far space). The second one is that LPA induces a significant modulation on spatial and temporal task, whereas RPA only acts on the temporal, but not on the spatial task. This pattern of results suggests that the effects induced by PA on representations of space and time are the same along the sagittal near-far axis, but different along the horizontal left-to-right axis. Therefore, we shall first discuss the results of PA modulation found in near and far space and then the asymmetrical effect of orienting spatial attention leftwards and rightward on space and time representations, and we shall finally try to understand the reason of such differences.

The finding that the attentional shift after adaptation, which occurred within the near space, may alter spatial-time representations in the two sectors of space in a similar fashion is the novelty of the study. Indeed, examining literature on prismatic adaptation, the visuospatial effects of this procedure in far space were investigated by Frassinetti and colleagues [59] in neglect patients and by Berberovic and Mattingley [60] in neurologically healthy individuals. As regards clinical population, the efficacy of RPA in alleviating spatial attentional symptoms in neglect patients who, following a lesion of the right hemisphere, showing a deficit in orienting attention to the contralesional hemispace, has been already demonstrated [61–64]. Frassinetti and coworkers [59] revealed that a training of two weeks with RPA induced a long-lasting amelioration of neglect symptoms both in the near and in the far spaces, as assessed by two different behavioral and ecological tasks. Specifically, to explore the presence of neglect in the far space a room description task was used, in which patients were asked to name the items (e.g., window and chair) displayed symmetrically on the left and on the right wall of a room (3.6 × 2.2 m). As a consequence of the beneficial effects of PA procedure, the number of omissions on the left side was reduced after the two weeks of training with RPA. As far as healthy individuals, Berberovic and Mattingley [60] found that left-shifting prisms induced a post-PA rightward shift on estimates of visual center for stimuli appearing in far space in the landmark task. Relevant to the present study, the same authors also showed a surprising rightward, instead of leftward modulation, as it would have been expected, in the far space only. Our data appear to be at odds with this previous finding. However, there are several substantial dissimilarities between the study by Berberovic and Mattingley [60] and our own that could potetially explain such a discordant result. One important difference concerns the experimental procedure and methods adopted. At odds with our study, in Berberovic and Mattingley's work both participants and screen were moved between blocks, line stimuli appeared displayed centrally on the screen, or displaced slightly to the left or right, and all the participants used bimanual responses in the landmark task. Moreover, half of the sample used the left hand, and half used the right hand in the adaptation session. We cannot therefore exclude that these procedural differences might have brought discrepant results. From a statistical point of view, and still contrary to our study, the data obtained from the landmark task in the Berberovic and Mattingley's study were analysed separately for the LPA and RPA groups through two one-way repeated-measures ANOVAs. More importantly, our sample is twice as large as that of Berberovic and Mattingley and this further allowed us to perform an omnibus MANOVA that we believe more powerful and suitable analysis for our study. Albeit differences in experimental procedures could have contributed to this different statistical result, we wish to emphasize that in a larger sample we did not replicate this finding. By visual inspection, in our sample there is a tendency towards a rightward shift in the far space, which does not reach significance.

Furthermore, as in Berberovic and Mattingley's study and previous studies [16, 28, 60], we found no linear correlation between the sensorimotor and cognitive aftereffects following adaptation. Indeed, it has already been established that the two aftereffects were uncorrelated in magnitude (i.e., the amount of sensorimotor aftereffect does not predict the amount of spatial modulation or its magnitude), and this finding is reminiscent of the lack of correlation between pointing errors and line bisection performance in neglect patients following prisms adaptation [28]. The lack of any significant relationship supports the idea that the changes found on the landmark and time bisection tasks are not directly related to the sensorimotor aftereffect. PA might indeed act at both sensorimotor and cognitive levels, but the link between the two different measures might not be a linear association and might imply more complex relationships.

Nevertheless, the finding that temporal and spatial changes following the procedure of adaptation are similar across representational spaces is in agreement with the hypothesis that PA-induced effects may be mediated by the oculomotor systems [65–67]. Indeed, during prismatic procedure participants perform a series of pointing movements, which relies on a form of visuomotor coordination between the hand and eye. Since movements are deviated toward the side of prismatic lenses, to compensate for the visual field displacement participants implicitly deviate their motor programs toward the left or right, thereby implementing a leftward or rightward recalibration into their sensorimotor systems. Due to the eye-hand coordination during pointing task, a deviation of eye movements in the direction opposite to the prismatic shift is expected. Provided that the areas involved in eye movements (area 8) and in eye movement programming (LIP) might contribute to far space representation [68, 69], the effects of PA in far space is compatible with a potential shift of the oculomotor system following the procedure of adaptation. Since the direction of eye movements can influence spatial representation [70], shifting oculomotor system can also impact the exploration on the horizontal mental time line, thus resulting in a modulation produced by PA on time representations, too, in near space as well as far space.

The second interesting result of the current study is that PA differently affects spatial and time processing, as indexed by comparing the landmark and the time bisection tasks. More in detail, LPA effects were found in landmark as well as in time bisection tasks, whereas RPA effects were limited to time bisection task. Indeed, in the spatial domain the effectiveness of PA in modulating spatial cognition has also been proven to be unidirectional in both pathological and neurologically healthy populations. Only RPA has been demonstrated to act on neglect symptoms [71] and, by contrast, only LPA has been so far reported to be effective in healthy individuals miming a neglect-like behavior. Accordingly, most of the studies investigating PA in healthy subjects did not find any significant plastic effects of RPA on spatial processing. Because of the lack of a significant leftward spatial modulation after rightward prism adaptation in nonclinical population, RPA has become the standard “control” procedure for studies examining LPA-induced effects on spatial processing. Moving on to a possible explanation of such a null effect, some authors [24] considered the null result regarding the effects of RPA in terms of a simulation of neglect in neurologically healthy individuals. Given that neglect syndrome is more likely to occur after right rather than left lesions, inducing a rightward bias of spatial attentional orientation, it has been put forward the hypothesis that the asymmetrical effects of LPA and RPA on spatial processing might reflect an inherent bias of the brain's structural organization in directing attention to the right [72]. Thus, some reports hint that neglect and pseudoneglect may share common cognitive and neural mechanisms, as they appear to be two sides of the same coin [19]. In other words, spatial performances of healthy participants after leftward prismatic adaptation could be considered as correct approximation of a neglect-like behavior, with common main characteristics (e.g., directional bias and directional specificity of PA [25]).

Moreover, regarding PA effects on time processing, our results are in line with previous findings in healthy subjects [11]. In fact, both PA directions symmetrically alter time processing and the directional bias observed after PA depends on the direction of the prismatic deviation. After rightward optical deviation inducing a leftward aftereffect, participants show a significant underestimation of perceived time, relative to before PA. Furthermore, after leftward optical deviation inducing a rightward aftereffect, participants report a significant overestimation of perceived time. Thus, results from time bisection task are consistent with the predictions of the anatomofunctional model of PA by Pisella and coworkers [73], but the results from the line bisection task are not. This model hypothesizes that PA may act by hypoactivating the posterior parietal cortex contralateral to the direction of the prismatic deviation and this, in turn, is able to modify the interhemispheric balance involved in orienting spatial attention. The model provides a solid theoretical framework for understanding the functioning of adaptation procedures because it evokes the same mechanism to explain both RPA's therapeutic effects in neglect (e.g., [59, 61]) and the induction of rightward (neglect-like) biases following LPA in healthy individuals (e.g., [16, 24]). According to this model, both prism directions are expected to modulate the contralateral posterior parietal cortex, and therefore both LPA and RPA should affect spatial processing in nonclinical population. In line with this account, here we stress the point that, although this symmetrical predicted modulation has not been demonstrated for the space domain yet, the expected effects are here found in the time domain, since we observed that leftward and rightward prism adaptation induced opposite plastic effects on time representations. Indeed, the finding that leftward- and rightward-deviating prisms had a different impact on intact spatial cognition was not an unexpected result. As mentioned above, it has been largely demonstrated that PA procedures have an asymmetric action on visuospatial cognition in both patients and healthy individuals. The same studies showing that LPA affects spatial processing in healthy participants have reported no effect of RPA [16, 24–27], even though the sensorimotor aftereffects (the hallmark of PA) induced by both LPA and RPA have comparable magnitudes and opposite directions. This negative evidence in the literature concerning the absence of RPA effects on intact spatial processing contrasts with the prediction of the anatomofunctional model proposed by Pisella and colleagues [73]. Here, we concur with the results of the previous studies, in that the model is not supported for the spatial domain. In spite of this, the prediction of the model is confirmed in temporal cognition, which has been shown to be directionally modulated by both PA directions, thus providing partial support to Pisella et al.'s model at least in the time domain.

**Figure 3:**
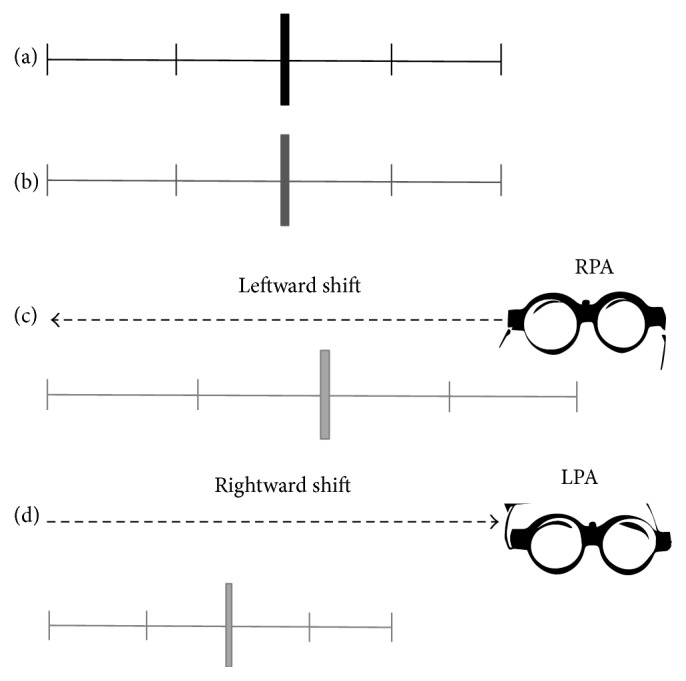
Theoretical model of the plastic prismatic adaptation effects on spatial representation of time processing. (a) The horizontal black line represents a putative duration to be bisected. The central vertical marker represents the real midpoint of the temporal duration. The little vertical lines represent time beats indicating the velocity of time flow passage: the greater the distance between the lines, the slower the passage of time flow. (b) The horizontal dark gray line represents time duration perceived by participants before PA. The central vertical marker represents the temporal bisection judgment of participants before PA. The real time duration and the perceived duration by participants are aligned: the passage of time flow of the perceived duration has the same velocity of the real duration. (c) Leftward shift of spatial attention following RPA induces an underestimation of time. The horizontal dashed black arrow represents the leftward shift of spatial attention induced by RPA. The horizontal light gray line represents the time duration perceived by participants after RPA. The central vertical marker represents the temporal bisection judgment of participants after RPA. The real time interval and the perceived interval are not aligned because of the rightward bias of spatial attention: the passage of time flow of the perceived duration beats more slowly than the real duration. (d) Rightward shift of spatial attention following LPA induces an overestimation of time. The horizontal dashed black arrow represents the leftward shift of spatial attention induced by LPA. The horizontal light gray line represents the time duration perceived by participants after LPA. The central vertical marker represents the temporal bisection judgment of participants after LPA. The real time interval and the perceived interval are not aligned because of the rightward bias of spatial attention: the passage of time flow of the perceived duration beats more quickly than the real duration.

Remarkably, these findings are also in accordance with the proposed PA's mechanism of action on time processing [74]. As posited by Oliveri et al.'s model, in the absence of manipulation of spatial attention, in healthy individuals real time and perceived time are aligned at the beginning of a temporal duration. The leftward shift of spatial attention via RPA, by shifting the spatial representation of the mental time line leftwards, produces a backward perception of elapsing time. Because of this bias, participants underestimate time durations: the passage of time flow of the perceived duration beats more slowly than the real duration. The same mechanism is proposed for the rightward shift of spatial attention after LPA. Because of the rightward shift of attention following LPA, time seems to speed up and participants overestimated time durations (see Figure 3).

Hence, we wonder why we did not find a similar symmetric modulation caused by the same attention manipulation on two so well-interrelated domains that are space and time. Although the exact nature of PA aftereffects is unclear, in order to explain the asymmetrical effects induced by PA procedure, we propose that temporal and spatial representations are not perfectly aligned, and thereby the attentional plastic changes caused by PA could enforce this different alignment between the two representations.

A simple approach for testing for the hypothesis of misalignment between spatial and temporal processing is provided by correlation analyses. It does not indeed appear to exist any consistent correlation between the spatial and temporal measures of the landmark and time bisection tasks. Note that correlation only looks at linear relationships, as Pearson's correlation is a measure of a linear association between two variables, whereas the nature of this cross-domain association may be much more complicated than a linear one. There may be a need for further and more focused research on this open issue.

Within this ever-growing interest for the understanding of these phenomenological dimensions, we directly assessed the plastic changes induced by a very well-known spatial attentional manipulation onto representations of time and space. Despite the lack of previous evidence in literatures comparing PA modulation in temporal and spatial processes, the reading of research from space-time interactions seems to suggest that, although tightly intertwined, the two domains may also differ in several respects. For instance, while most physical quantities can be represented in an ascending and a descending manner, perceived time always runs in the same direction (the anisotropy of time). Along the same lines, whereas time is therefore one-dimensional, space is instead three-dimensional. Admittedly, when talking about time, one should take into account that our understanding of this domain could go far beyond simple spatially based concepts of duration according to a specific direction being ascending from left to right [75]. Time encompasses many different aspects, including, for example, ordinality (e.g., what comes before versus after) and sequentiality (e.g., first versus second) or historical aspects (e.g., 1960s versus 1980s) [76]. Some of these proprieties of time might rely on a left-to-right horizontally oriented spatial representation [9, 77–79], but other aspects might not. Some scholars have also found evidence for the cognitive reality of front-back spatial representation of time with the past mapping to the back and the future mapping to the front [80]. Furthermore, the iteration of day and night and yearly seasons is not captured by the mental time line, so that some cultures around the world have developed “cyclic” time models. Additionally, we can use complementary, multiple spatial metaphors to understand the elapsed time and temporal relations, considering that time is not a monolith, but rather a mosaic of constructs (for a review [81]). Consistent with this view, even though the domains of space and time are closely interconnected and rely on shared neural resources, they also involve distinct structures and circuits. ATOM does indeed propose a shared neural substrate for representing and manipulating magnitudes but also acknowledges that the representation of all magnitudes may involve additional domain-specific neural substrates [2]. Along the same line of reasoning, ATOM's supporters proposed that “all magnitudes are not created equal” [2]. Indeed, interactions between space and time may be asymmetrical, such that, for instance, spatial cues may have a larger effect on temporal judgments than temporal cues do on spatial judgments [82]. There is considerable research demonstrating selective influences of the domain of space on the domain of time, but not corresponding influences in the other direction [75, 81, 83, 84]. So, although the ATOM framework does not explicitly predict spatial-temporal asymmetrical interactions, the model is in principle compatible with asymmetries that derive from differences in the domains themselves [2]. For all these reasons, at the moment, it is premature to provide a definitive account of the cross-domain temporal-spatial interactions given the complexity of each phenomenology of space and time.

## 5. Conclusions

Coming back to the purpose of the present study that is to directly compare spatial and temporal alterations after PA in the near and the far space, here we underline the two main findings. The first one is that PA acts in a similar fashion on spatial and temporal processing of stimuli on the sagittal axis (near/far). The second one is that the shift of attention along the horizontal axis (left/right) differently affects space and time. In this respect, it is important to note a difference when we consider sagittal near/far axis and horizontal left/right axis. Indeed, near and far are referred to as a* physical* distance from the body, whereas left and right are referred to as a* mental* spatial-temporal representation. Once prismatic adaptation has acted on this spatial-temporal representation, thus inducing different effects on space and time dimensions according to leftward/rightward attentional shift, the changes due to this manipulation may be extended to all the physical distances.

## References

[1] Walsh V. (2003). A theory of magnitude: common cortical metrics of time, space and quantity. *Trends in Cognitive Sciences*.

[2] Bueti D., Walsh V. (2009). The parietal cortex and the representation of time, space, number and other magnitudes. *Philosophical Transactions of the Royal Society B: Biological Sciences*.

[3] Ishihara M., Keller P. E., Rossetti Y., Prinz W. (2008). Horizontal spatial representations of time: evidence for the STEARC effect. *Cortex*.

[4] Arzy S., Adi-Japha E., Blanke O. (2009). The mental time line: an analogue of the mental number line in the mapping of life events. *Consciousness and Cognition*.

[5] Santiago J., Lupáñez J., Pérez E., Funes M. J. (2007). Time (also) flies from left to right. *Psychonomic Bulletin & Review*.

[6] Torralbo A., Santiago J., Lupiáñez J. (2006). Flexible conceptual projection of time onto spatial frames of reference. *Cognitive Science*.

[7] Vallesi A., Weisblatt Y., Semenza C., Shaki S. (2014). Cultural modulations of space-time compatibility effects. *Psychonomic Bulletin and Review*.

[8] Oliveri M., Koch G., Salerno S., Torriero S., Gerfo E. L., Caltagirone C. (2009). Representation of time intervals in the right posterior parietal cortex: Implications for a mental time line. *NeuroImage*.

[9] Di Bono M. G., Casarotti M., Priftis K., Gava L., Umiltà C., Zorzi M. (2012). Priming the mental time line. *Journal of Experimental Psychology: Human Perception and Performance*.

[10] Vicario C. M., Pecoraro P., Turriziani P., Koch G., Caltagirone C., Oliveri M. (2008). Relativistic compression and expansion of experiential time in the left and right space. *PLoS ONE*.

[11] Frassinetti F., Magnani B., Oliveri M. (2009). Prismatic lenses shift time perception. *Psychological Science*.

[12] Gaveau V., Prablanc C., Laurent D., Rossetti Y., Priot A.-E. (2014). Visuomotor adaptation needs a validation of prediction error by feedback error. *Frontiers in Human Neuroscience*.

[13] Redding G. M., Wallace B. (2013). Prism adaptation in alternately exposed hands. *Attention, Perception, and Psychophysics*.

[14] Bultitude J. H., Van der Stigchel S., Nijboer T. C. W. (2013). Prism adaptation alters spatial remapping in healthy individuals: evidence from double-step saccades. *Cortex*.

[15] Redding G. M., Rossetti Y., Wallace B. (2005). Applications of prism adaptation: a tutorial in theory and method. *Neuroscience and Biobehavioral Reviews*.

[16] Schintu S., Pisella L., Jacobs S., Salemme R., Reilly K. T., Farnè A. (2014). Prism adaptation in the healthy brain: the shift in line bisection judgments is long lasting and fluctuates. *Neuropsychologia*.

[17] Magnani B., Oliveri M., Renata Mangano G., Frassinetti F. (2010). The role of posterior parietal cortex in spatial representation of time: a TMS study. *Behavioural Neurology*.

[18] Magnani B., Pavani F., Frassinetti F. (2012). Changing auditory time with prismatic goggles. *Cognition*.

[19] Jewell G., McCourt M. E. (2000). Pseudoneglect: a review and meta-analysis of performance factors in line bisection tasks. *Neuropsychologia*.

[20] Schmitz R., Peigneux P. (2011). Age-related changes in visual pseudoneglect. *Brain and Cognition*.

[21] Toba M.-N., Cavanagh P., Bartolomeo P. (2011). Attention biases the perceived midpoint of horizontal lines. *Neuropsychologia*.

[22] Benwell C. S. Y., Thut G., Grant A., Harvey M. (2014). A rightward shift in the visuospatial attention vector with healthy aging. *Frontiers in Aging Neuroscience*.

[23] Longo M. R., Trippier S., Vagnoni E., Lourenco S. F. (2015). Right hemisphere control of visuospatial attention in near space. *Neuropsychologia*.

[24] Colent C., Pisella L., Bernieri C., Rode G., Rossetti Y. (2000). Cognitive bias induced by visuo-motor adaptation to prisms: a simulation of unilateral neglect in normal individuals?. *NeuroReport*.

[25] Michel C., Pisella L., Halligan P. W. (2003). Simulating unilateral neglect in normals using prism adaptation: implications for theory. *Neuropsychologia*.

[26] Loftus A. M., Vijayakumar N., Nicholls M. E. R. (2009). Prism adaptation overcomes pseudoneglect for the greyscales task. *Cortex*.

[27] Nijboer T., Vree A., Dijkerman C., Van der Stigchel S. (2010). Prism adaptation influences perception but not attention: evidence from antisaccades. *NeuroReport*.

[28] Pisella L., Rode G., Farnè A., Boisson D., Rossetti Y. (2002). Dissociated long lasting improvements of straight-ahead pointing and line bisection tasks in two hemineglect patients. *Neuropsychologia*.

[29] Reed S. A., Dassonville P. (2014). Adaptation to leftward-shifting prisms enhances local processing in healthy individuals. *Neuropsychologia*.

[30] Ferrè E. R., Longo M. R., Fiori F., Haggard P. (2013). Vestibular modulation of spatial perception. *Frontiers in Human Neuroscience*.

[31] Gamberini L., Seraglia B., Priftis K. (2008). Processing of peripersonal and extrapersonal space using tools: evidence from visual line bisection in real and virtual environments. *Neuropsychologia*.

[32] Lourenco S. F., Longo M. R. (2009). The plasticity of near space: evidence for contraction. *Cognition*.

[33] Longo M. R., Lourenco S. F. (2006). On the nature of near space: effects of tool use and the transition to far space. *Neuropsychologia*.

[34] Longo M. R., Lourenco S. F. (2007). Space perception and body morphology: extent of near space scales with arm length. *Experimental Brain Research*.

[35] Berti A., Frassinetti F. (2000). When far becomes near: remapping of space by tool use. *Journal of Cognitive Neuroscience*.

[36] Witt J. K., Proffitt D. R., Epstein W. (2005). Tool use affects perceived distance, but only when you intend to use it. *Journal of Experimental Psychology: Human Perception and Performance*.

[37] Cardinali L., Frassinetti F., Brozzoli C., Urquizar C., Roy A. C., Farnè A. (2009). Tool-use induces morphological updating of the body schema. *Current Biology*.

[38] Makin T. R., Brozzoli C., Cardinali L., Holmes N. P., Farnè A. (2015). Left or right? Rapid visuomotor coding of hand laterality during motor decisions. *Cortex*.

[39] Cardellicchio P., Sinigaglia C., Costantini M. (2011). The space of affordances: a TMS study. *Neuropsychologia*.

[40] Makin T. R., Holmes N. P., Brozzoli C., Rossetti Y., Farnè A. (2009). Coding of visual space during motor preparation: approaching objects rapidly modulate corticospinal excitability in hand-centered coordinates. *Journal of Neuroscience*.

[41] Serino A., Annella L., Avenanti A. (2009). Motor properties of peripersonal space in humans. *PLoS ONE*.

[42] Farnè A., Làdavas E. (2000). Dynamic size-change of hand peripersonal space following tool use. *NeuroReport*.

[43] Maravita A., Husain M., Clarke K., Driver J. (2001). Reaching with a tool extends visual-tactile interactions into far space: evidence from cross-modal extinction. *Neuropsychologia*.

[44] Costantini M., Frassinetti F., Maini M., Ambrosini E., Gallese V., Sinigaglia C. (2014). When a laser pen becomes a stick: remapping of space by tool-use observation in hemispatial neglect. *Experimental Brain Research*.

[45] Makin T. R., Holmes N. P., Zohary E. (2007). Is that near my hand? Multisensory representation of peripersonal space in human intraparietal sulcus. *The Journal of Neuroscience*.

[46] Brozzoli C., Gentile G., Petkova V. I., Ehrsson H. H. (2011). fMRI adaptation reveals a cortical mechanism for the coding of space near the hand. *Journal of Neuroscience*.

[47] Brozzoli C., Gentile G., Henrik Ehrsson H. (2012). That's near my hand! Parietal and premotor coding of hand-centered space contributes to localization and self-attribution of the hand. *Journal of Neuroscience*.

[48] Brozzoli C., Gentile G., Bergouignan L., Ehrsson H. H. (2013). A shared representation of the space near oneself and others in the human premotor cortex. *Current Biology*.

[49] Cléry J., Guipponi O., Wardak C., Ben Hamed S. (2015). Neuronal bases of peripersonal and extrapersonal spaces, their plasticity and their dynamics: knowns and unknowns. *Neuropsychologia*.

[50] di Pellegrino G., Làdavas E. (2015). Peripersonal space in the brain. *Neuropsychologia*.

[51] Brozzoli C., Ehrsson H., Farnè A. (2014). Multisensory representation of the space near the hand: from perception to action and interindividual interactions. *The Neuroscientist*.

[52] Milner A. D., Brechmann M., Pagliarini L. (1992). To halve and to halve not: an analysis of line bisection judgements in normal subjects. *Neuropsychologia*.

[53] McCourt M. E., Olafson C. (1997). Cognitive and perceptual influences on visual line bisection: psychophysical and chronometric analyses of pseudoneglect. *Neuropsychologia*.

[54] Olk B., Harvey M. (2002). Effects of visible and invisible cueing on line bisection and Landmark performance in hemispatial neglect. *Neuropsychologia*.

[55] Kliegl K. M., Limbrecht-Ecklundt K., Dürr L., Traue H. C., Huckauf A. (2015). The complex duration perception of emotional faces: effects of face direction. *Frontiers in Psychology*.

[56] Lindbergh C. A., Kieffaber P. D. (2013). The neural correlates of temporal judgments in the duration bisection task. *Neuropsychologia*.

[57] Suarez I., Lopera F., Pineda D., Casini L. (2013). The cognitive structure of time estimation impairments in adults with attention deficit hyperactivity disorder. *Cognitive Neuropsychology*.

[58] Oldfield R. C. (1971). The assessment and analysis of handedness: the Edinburgh inventory. *Neuropsychologia*.

[59] Frassinetti F., Angeli V., Meneghello F., Avanzi S., Làdavas E. (2002). Long-lasting amelioration of visuospatial neglect by prism adaptation. *Brain*.

[60] Berberovic N., Mattingley J. B. (2003). Effects of prismatic adaptation on judgements of spatial extent in peripersonal and extrapersonal space. *Neuropsychologia*.

[61] Rossetti Y., Rode G., Pisella L. (1998). Prism adaptation to a rightward optical deviation rehabilitates left hemispatial neglect. *Nature*.

[62] Farnè A., Rossetti Y., Toniolo S., Làdavas E. (2002). Ameliorating neglect with prism adaptation: visuo-manual and visuo-verbal measures. *Neuropsychologia*.

[63] Striemer C. L., Danckert J. (2010). Dissociating perceptual and motor effects of prism adaptation in neglect. *NeuroReport*.

[64] Newport R., Schenk T. (2012). Prisms and neglect: what have we learned?. *Neuropsychologia*.

[65] Angeli V., Benassi M. G., Làdavas E. (2004). Recovery of oculo-motor bias in neglect patients after prism adaptation. *Neuropsychologia*.

[66] Serino A., Angeli V., Frassinetti F., Làdavas E. (2006). Mechanisms underlying neglect recovery after prism adaptation. *Neuropsychologia*.

[67] Serino A., Barbiani M., Rinaldesi M. L., Ladavas E. (2009). Effectiveness of prism adaptation in neglect rehabilitation: a controlled trial study. *Stroke*.

[68] Rizzolatti G., Gallese V., Boller F., Grafman J. (1988). Mechanisms and theories of spatial neglect. *Handbook of Neuropsychology*.

[69] Colby C. L., Duhamel J.-R., Goldberg M. E. (1996). Visual, presaccadic, and cognitive activation of single neurons in monkey lateral intraparietal area. *Journal of Neurophysiology*.

[70] Meador K. J., Loring D. W., Bowers D., Heilman K. M. (1987). Remote memory and neglect syndrome. *Neurology*.

[71] Luauté J., Jacquin-Courtois S., O'Shea J. (2012). Left-deviating prism adaptation in left neglect patient: reflexions on a negative result. *Neural Plasticity*.

[72] Goedert K. M., Leblanc A., Tsai S.-W., Barrett A. M. (2010). Asymmetrical effects of adaptation to left- and right-shifting prisms depends on pre-existing attentional biases. *Journal of the International Neuropsychological Society*.

[73] Pisella L., Rode G., Farnè A., Tilikete C., Rossetti Y. (2006). Prism adaptation in the rehabilitation of patients with visuo-spatial cognitive disorders. *Current Opinion in Neurology*.

[74] Oliveri M., Magnani B., Filipelli A., Avanzi S., Frassinetti F. (2013). Prismatic adaptation effects on spatial representation of time in neglect patients. *Cortex*.

[75] Winter B., Marghetis T., Matlock T. (2015). Of magnitudes and metaphors: explaining cognitive interactions between space, time, and number. *Cortex*.

[76] Masson N., Pesenti M., Dormal V. (2015). Duration and numerical estimation in right brain-damaged patients with and without neglect: lack of support for a mental time line. *British Journal of Psychology*.

[77] Saj A., Fuhrman O., Vuilleumier P., Boroditsky L. (2014). Patients with left spatial neglect also neglect the ‘left side’ of time. *Psychological Science*.

[78] Weger U. W., Pratt J. (2008). Time flies like an arrow: space-time compatibility effects suggest the use of a mental timeline. *Psychonomic Bulletin & Review*.

[79] Bonato M., Zorzi M., Umiltà C. (2012). When time is space: evidence for a mental time line. *Neuroscience & Biobehavioral Reviews*.

[80] Núñez R. E., Sweetser E. (2006). With the future behind them: convergent evidence from Aymara language and gesture in the crosslinguistic comparison of spatial construals of time. *Cognitive Science*.

[81] Núñez R., Cooperrider K. (2013). The tangle of space and time in human cognition. *Trends in Cognitive Sciences*.

[82] Casasanto D., Boroditsky L. (2008). Time in the mind: using space to think about time. *Cognition*.

[83] Bottini R., Casasanto D. (2013). Space and time in the child's mind: metaphoric or ATOMic. *Frontiers in Psychology*.

[84] Casasanto D., Fotakopoulou O., Boroditsky L. (2010). Space and time in the child's mind: evidence for a cross-dimensional asymmetry. *Cognitive Science*.

